# Implementation Science for Emergency Physicians: A Schematic Diagram and Step-By-Step Guidance to Implement Evidence-Based Innovations

**DOI:** 10.1016/j.acepjo.2026.100501

**Published:** 2026-08-27

**Authors:** Emily S. Fu, Linda E. Guzman, Yosef Berlyand, Stephanie Chow Garbern, Debasree Banerjee, Kristine Durkin, Hannah E. Frank

**Affiliations:** 1 Departments of Medicine and Psychiatry and Behavioral Neuroscience, The University of Chicago, Chicago, Illinois, USA; 2 Department of Psychiatry and Human Behavior, The Warren Alpert Medical School of Brown University, Providence, Rhode Island, USA; 3 Department of Emergency Medicine, The Warren Alpert Medical School of Brown University, Providence, Rhode Island, USA; 4 Department of Medicine, The Warren Alpert Medical School of Brown University, Providence, Rhode Island, USA

## Abstract

Emergency physicians are pivotal in ensuring that patients seeking urgent medical care for various illnesses receive evidence-based care. However, there can be variable adherence to evidence-based care on the individual, interpersonal, and institutional levels. Emergency medicine settings may benefit from using implementation science theories, models, and frameworks to introduce new guidelines and recommendations to increase adherence and uptake of evidence-based protocols. At the same time, implementation science applications must consider the unique context of the emergency medicine setting. The authors of this paper are a multidisciplinary group of emergency physicians, behavioral researchers, and implementation scientists, who identified a need for emergency physicians to have practical implementation guidance. During a 3-day implementation science workshop, authors collaboratively developed a schematic diagram, a step-by-step guide, and a case example, presented here, to guide the implementation of evidence-based practices in the emergency department. In summary, this paper aims to make knowledge translation accessible to emergency physicians by presenting a resource for practical implementation science applications.

## 1 Background

Emergency physicians (EPs) face the challenge of remaining up to date with the latest medical research and practice guidelines^1^^,^^2^ while working in highly complex, time-pressured environments. Workflow challenges, staffing limitations, and complex care coordination make implementation of evidence-based innovations (EBIs) particularly difficult in emergency departments (EDs)^3^^,^^4^ and may result in overreliance on ingrained clinical practice. Moreover, ED team composition is highly variable, as clinicians and staff are assembled in different combinations across shifts and from day to day.^5^ Although initiatives, such as external incentive systems, aim to change practice patterns, prior studies demonstrate substantial practice pattern variation, poor adherence to best practice protocols, and variable uptake of EBIs in EDs for conditions, such as sepsis, low-risk chest pain, pneumonia, and hypertensive emergencies.^6^^,^^7^ For example, in 2024, the Centers for Medicare & Medicaid Services (CMS) mandated inpatient screening of Social Determinants of Health (SDOH). Between 2019 and 2023, only 0.2% (of *n* = 383,596) ED encounters screened for SDOH, highlighting the need for significant changes to successfully incorporate the mandate.^8^ In response, Loo et al^9^ interviewed 27 ED leaders in the United States to examine barriers and facilitators to SDOH screening. ED leaders reported mixed attitudes, including skepticism about the utility of ED SDOH screening, and challenges related to resources, staffing, and insufficient time. This underscores the need to incorporate targeted strategies to address barriers to implementation.

The field of implementation science is well-positioned to address barriers to the uptake of EBIs, such as SDOH screening in EDs. Implementation science is “…the scientific study of methods to promote the systemic uptake of research findings and other EBIs into routine practice, and hence, to improve the quality and effectiveness of health services”.^10^ Such EBIs can be organized into seven categories, which can be delivered in different contexts, including the ED setting: Programs, Practices, Principles, Procedures, Products, Pills and Policies.^11^ However, few resources provide guidance for implementing EBIs, including responses to mandates, in the ED setting. In 2018, de Wit et al^12^ published 10 recommendations for best implementation practices for Canadian emergency departments. A systematic review by Ebben et al^13^ included 11 studies to evaluate the effectiveness of implementation strategies for improving guideline and protocol adherence in ED settings. The authors exhibited that educational interventions, reminders, and multifaceted approaches such as audit and feedback showed promise for improving guideline and protocol adherence in emergency care settings. Indeed, Beryland et al^14^ used a multifaceted approach at a medical center to increase use of sepsis order sets. However, given the limited number of eligible studies, authors were unable to draw firm conclusions, and implementation outcomes were infrequently measured. Furthermore, implementation strategies vary in effectiveness by site and require rigorous implementation science methods to identify and carry out. Together, these findings highlight the limited number of implementation studies in the ED setting and the need for clearer guidance on how EP researchers can use implementation science to improve EBI implementation within their unique ED contexts.

## 2 Objectives

This paper provides high-level, step-by-step guidance for applying seminal implementation science theories, models, and frameworks to assist EPs in implementing EBIs. The paper was developed through a collaboration between physicians in emergency and critical care settings, implementation scientists, and behavioral researchers. During a multiday implementation science workshop at an academic medical center, the physicians expressed a need to clarify the practical application of implementation science methods for emergency medicine. Thus, the paper’s schematic diagram and four-step process were developed based on the workshop’s content to: (1) guide the development of implementation studies for EPs and (2) be used as an implementation practice tool, complementary to quality improvement (QI) initiatives.

**Case Example: Implementing a Sepsis Response Team**

To demonstrate each step of the process, this article presents a case example about an EP investigator-led initiative to implement a multidisciplinary sepsis response team in the ED. Sepsis continues to be the leading cause of Intensive Care Unit (ICU) admissions and fatalities and is the most common cause of inhospital mortality in the United States.^15^ In response, a group of EP investigators at a health system in Rhode Island are using implementation science methods to implement a *Code Sepsis* workflow, or “procedure” according to the 7Ps, to improve timely recognition of sepsis.^16^ *Code Sepsis* focuses on implementing the clinically important and guideline-supported components of early sepsis care, especially early recognition, timely antibiotics for patients with probable sepsis/septic shock, and coordinated team-based response. The case example will illustrate how the 4-step process is applied to design and evaluate the implementation of this real-world project.

**A Step-by-Step Process for Applying Implementation Science**

**Figure 1:**
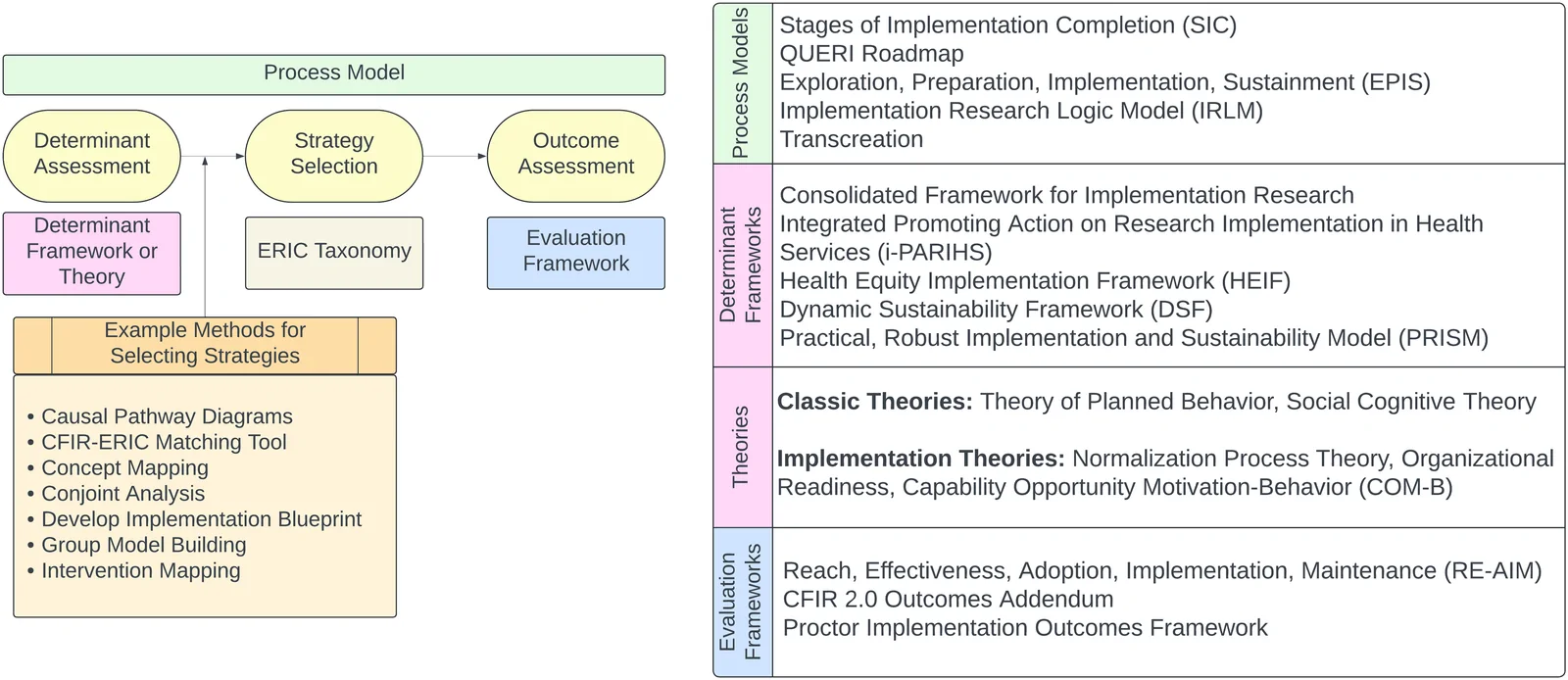
The schematic diagram for designing implementation studies for emergency physician researchers.

Theories, Models, and Frameworks (TMFs) are central to designing implementation projects. TMFs are typically divided into 3 categories, each of which serves a different function.^17^: (1) **Process models** to describe and/or guide the implementation; (2) **determinant frameworks,** and **classic and implementation theories** to understand what factors and mechanisms influence implementation outcomes; (3) **evaluation frameworks** to measure implementation outcomes. The relationship between each type of TMFs is displayed in the schematic diagram (Fig 1). The goal of this schematic diagram (provided in template form in Appendix 1) is to help guide the design of implementation research and practice efforts, and to highlight how TMFs may help carry out existing QI initiatives. The following sections illustrate the steps of using Figure 1. First, the section describes how process models can be selected and applied to guide program implementation. Second, determinant frameworks and ways they are used to identify barriers and facilitators to program implementation are introduced. Third, the selection and operationalization of implementation strategies to address identified barriers are discussed. Fourth, the measurement of implementation outcomes using evaluation frameworks is explained.

**Step 1: Select a Process Model**

The primary function of process models is to outline the stages of implementation and provide concrete steps involved in carrying out program implementation. This section describes 3 examples of commonly used process models and how they can guide implementation.

1) The 4-phase *Exploration, Preparation, Implementation, and Sustainment (EPIS)* is one of the most commonly used process models.^18^ The purpose of the *Exploration* phase is to assess the need and fit of a program in the ED setting. This may involve evaluating an organization’s implementation culture, climate, and leadership, as well as individual perceptions of the EBI. The *Exploration* phase is typically carried out using a *Determinant Framework* (see Step 2). Next, the *Preparation* phase entails planning for program introduction to enhance program uptake. The *Implementation* phase focuses on introducing and monitoring the program and examining barriers and facilitators to the program. The *Sustainment* phase evaluates the maintenance of an EBI in a service setting after the research period ends.
2) *Stages of Implementation Completion (SIC)* is a process model that divides implementation activities into preimplementation, implementation, and sustainment stages, each with activities that are central to carrying out and completing each stage.^19^ The SIC is a date-based measure in which researchers input the date each implementation activity is completed. The dates are used to calculate the pace of implementation (e.g., how many days it took to complete each stage) and the percentage of activities completed in each stage of implementation.^20^
3) The *Implementation Research Logic Model (IRLM)* is a process model that assists with designing for implementation, enhancing relationships between constructs, and rigorously organizing implementation projects for ease of reproducibility.^21^ The IRLM has 4 sequential parts, including: 1) implementation determinants, 2) strategies to address the determinants, 3) mechanisms (i.e., how and why the implementation strategies work to address the determinants), and 4) outcomes.

To explore more options, readers can refer to dissemination-implementation.org, an interactive, online resource designed to help researchers and practitioners navigate TMFs.

**Case example: Selection of a Process Model**

The IRLM was selected as the process model for the *Code Sepsis* project because it allows researchers to link a determinant framework with mechanisms, outcomes, and strategies, capturing the whole implementation process in one model. After selecting the IRLM as the process model, the next step is to select a determinant framework to assess the barriers to implementing *Code Sepsis* in the ED setting.

**Step 2: Select a Determinant Framework to Conduct a Determinant Assessment**

The goal of the determinant assessment is to understand factors, often categorized as barriers or facilitators, that influence how well the intervention will be implemented.^17^ Although barriers are often emphasized because they require planning to overcome, facilitators (eg, strong leadership support, staff motivation) are equally important and can be leveraged to support implementation. Determinant assessments typically consider multiple ecological levels, such as individual attitudes, clinic resources, and policy considerations. Information can be collected through multiple methods, from quantitative surveys with key personnel to rigorous qualitative approaches, such as semistructured interviews, focus groups, or direct workflow observations. Interviews are particularly important in ED settings, where workflow complexity and time constraints may not fully be captured through surveys alone. For example, electronic health record alerts are commonly used to address implementation barriers. However, qualitative evaluations have revealed that clinicians tend to ignore the alerts because they occur too late in the workflow or contribute to alert fatigue.^22^ Thus, incorporating qualitative methods can better identify determinants in the ED setting.

Determinant assessments may occur at any time in the implementation process (i.e., before, during, or after implementation). Determinant assessments are best organized with a *determinant framework*. Although classic and implementation theories explain mechanisms underlying behavior change, determinant frameworks are more commonly used because they include barriers and facilitators across individual, organizational, and policy domains.^17^ Choosing a determinant framework depends on the specific goals and context of the implementation effort. One widely used framework is the updated Consolidated Framework for Implementation Research (CFIR 2.0).^23^ The CFIR 2.0 includes 5 domains: *Individuals* (eg, providers/staff delivering the intervention, patients receiving the intervention), *inner setting* (eg, department culture, incentive systems), *outer setting* (eg, policies and law, systemic conditions), *characteristics of the innovation*, and the *implementation process* (eg, tailoring strategies). Another determinant framework is the Theoretical Domains Framework (TDF),^18^ which focuses on factors influencing individual behavior change, such as what people know, what they believe they can do, and what factors help or hinder change.

Determinant frameworks can complement QI efforts, which are likely already occurring in EDs. QI approaches are commonly used in health care settings to improve existing processes, systems, and services to achieve better outcomes.^24^ Determinant frameworks can help systematically analyze and organize QI feedback in a meaningful way, and further study how to increase program use through identifying barriers and facilitators. Thus, the 2 approaches are inherently complementary. Beidas et al.^25^ have proposed a taxonomy of implementation science–QI integration models, ranging from QI-led efforts supported by implementation science tools to equal partnerships between Implementation science and QI. The pragmatic implementation science schematic presented here is intended to be used in combination with QI methods that may also focus on identifying and addressing implementation barriers.

Determinant assessments often result in more barriers than a project can address. Thus, a method to prioritize barriers is recommended before moving to Step 3. Generally, key informants rate the barriers among dimensions that are deemed most valuable to the system implementing the intervention (eg, cost, importance; see Prioritizing Implementation Barriers Toolkit). The barriers that are rated most highly are typically the focus of initial implementation efforts. For example, Frank et al.^26^ led a project that aimed to improve the implementation and adoption of a produce prescription program. After conducting CFIR 2.0-guided determinant assessments, the implementation science team and a community advisory board met for multiple meetings to rate barriers according to: 1) their impact and 2) if they could be feasibly addressed with the available time and resources.

**Case example: Conducting the Determinant Assessment for *Code Sepsis***

**Figure 2:**
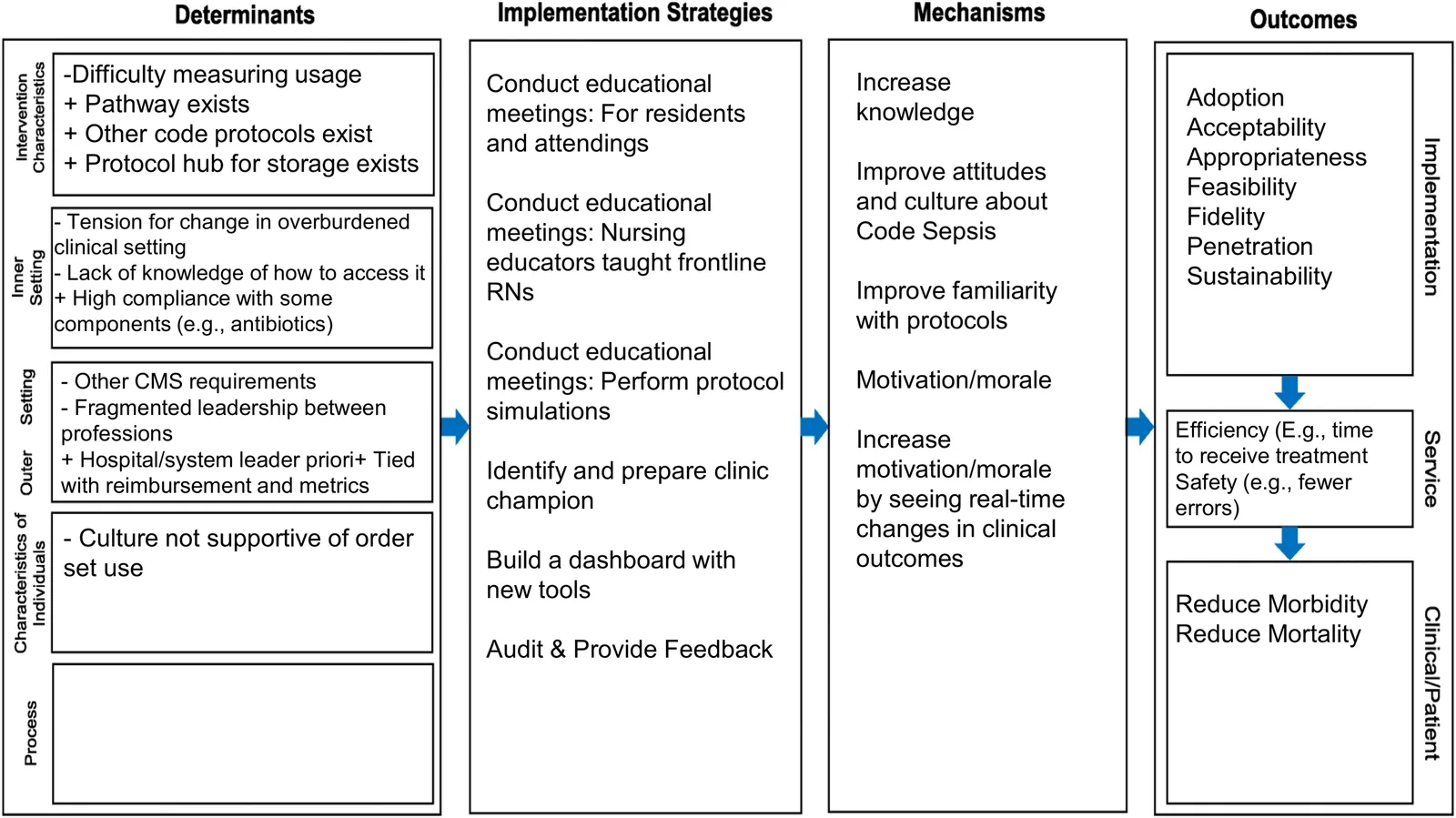
The complete implementation research logic model for the Code Sepsis project.

The research team sought multilevel feedback about factors that facilitated and hindered the adoption of *Code Sepsis*. The CFIR 2.0 framework informed the interview questions and organized the responses. The *Code Sepsis* investigators brought together key informants from the hospital’s physician quality leadership, physician medical directors, nursing quality leadership, and nursing educators. The meetings sought to collect multidisciplinary input about implementing *Code Sepsis* and assess the informants’ openness/resistance to change in general and regarding the use of sepsis bundles. All barriers, organized by CFIR 2.0, are listed in the first column of the *Code Sepsis* IRLM in Figure 2.

**Step 3: Choose a Method and/or Taxonomy to Select, Specify, and Operationalize Implementation Strategies**

Once barriers have been identified, the next step is to select implementation strategies to address the barriers. Implementation strategies are actions to overcome implementation barriers and harness facilitators.^27^ Strategies can be newly developed or based on the Expert Recommendations for Implementing Change (ERIC) taxonomy of 73 implementation strategies,^28^ which were developed and refined through a modified Delphi process involving expert consensus among implementation scientists.

The purpose of doing a determinant assessment and barrier prioritization (step 2) is to ensure that selected barriers, if addressed, will most likely lead to meaningful change. In step 3, barriers are collaboratively matched with potentially effective strategies. Continued engagement in conversations with key informants or a similar group can ensure alignment between the selection of strategies and their ability to address priority barriers.

Multiple methods can be used to ensure that selected strategies align with identified barriers. Some of these require specialized knowledge of methods like concept mapping^29^ and group model building. Other methods may be more pragmatic, such as the CFIR-ERIC matching tool.^30^ When using this tool, the CFIR-identified determinants (from Step 2) are matched with strategies from the ERIC taxonomy. This can be done through collaborative conversations with key informants about what strategies would effectively address the prioritized barriers. Causal pathway diagramming^31^ is another method that is well suited to ensure alignment between addressing barriers and selecting appropriate implementation strategies. Causal pathway diagrams are a way to visualize identified barriers, their corresponding mechanisms, and strategies that engage mechanisms and overcome barriers. The IMPACT Center provides a toolkit with step-by-step guidance on using causal pathway diagrams.^32^ Regardless of what approach is used, it is recommended that selection of the ERIC strategies is based on consensus with key informants. After strategies are selected, they must be specified and operationalized, so they are reproducible.^27^ The researcher or project leader should name the strategy and define any discrete components. For example, if the strategy is to distribute educational materials, the type of materials should be specified (eg, posters or emails), as should the recipients (eg, providers or leaders) and timeframe (e.g., a specific month or frequency). For research studies, reporting guidelines (eg, Proctor et al, 2013;^27^ Rudd et al, 2020^33^) require additional specifications to improve replicability of implementation strategies across studies.

**Case example: Strategy Selection and Operationalization**

After completing the determinant assessment, the EP investigator and key informants met for multiple meetings to discuss mechanisms that contribute to the identified barriers. Next, they chose strategies to address the barriers using the CFIR-ERIC Strategy Matching tool. After the strategies were selected, the investigators and key informants operationalized each strategy, described below. Table 1 shows how each strategy was operationalized according to Proctor et al,^27^ strategy specification criteria. Furthermore, the 2^nd^ and 3^rd^ columns of the *Code Sepsis* IRLM in Figure 2, show how the determinants informed the implementation strategies, and which mechanisms the strategies were designed to address.

**Table 1:** Operationalizing *Code Sepsis* implementation strategies according to Proctor et al. (2011)

Name it	Define it	Specify it The actor The action Action target Temporality, dose, implementation outcome affected, justification
Identify and prepare clinician champion (ERIC strategy)	Identify and prepare individuals who dedicate themselves to supporting Code Sepsis, and assist the organization in overcoming indifference or resistance	Actors: Study PI and Clinical leader Action: Identify and train clinical champion(s) within the ED department Action target: Inner setting attitudes, culture Temporality: Implementation phase Dose: 3 training meetings, monthly check-ins between champions and PIs Implementation outcome affected: Acceptability, Appropriateness, Adoption, Fidelity Justification: Reinforce knowledge and support of the *Code Sepsis* protocols within the department
Conduct educational meetings (ERIC strategy)	Hold meetings targeted toward residents and physicians to teach them about the clinical innovation	Actor: study PI and Department physician quality leader Action: present general sepsis and *Code Sepsis* education to residents and physicians, email follow-up information Action target: knowledge about sepsis and Code Sepsis, attitudes about *Code Sepsis* Temporality: Implementation phase, when incoming interns start Dose: Two presentations, weekly emails Implementation outcome affected: Adoption, Acceptability, Appropriateness, Feasibility, Penetration Justification: To improve knowledge and attitudes about *Code Sepsis* using existing clinic meetings
Conduct educational meetings (ERIC strategy)	Hold meetings targeted toward registered nurses to teach them about the clinical innovation	Actor: RN nursing educators Action: Present about *Code Sepsis* at regular education huddles Action target: attitudes, familiarity Temporality: Implementation phase Dose: daily for two weeks Implementation outcome affected: Adoption, Acceptability, Appropriateness, Feasibility, Penetration Justification: To improve knowledge and attitudes about *Code Sepsis*
Conduct educational meetings (ERIC strategy)	Provide specific, clear guidance about how to carry-out Code Sepsis procedures	Actor: RN nursing educators Action: Conduct *Code Sepsis* simulations with the RNs Action target: Attitudes, skills, familiarity Temporality: Implementation Dose: 2 simulations Implementation outcome affected: Adoption, Feasibility, Fidelity, Penetration Justification: Reinforce knowledge of the Code Sepsis protocols during existing meetings
Develop tools for quality monitoring (ERIC strategy)	Collaborate with hospital system leadership to build a dashboard with new tools to capture Code Sepsis activations in the Electronic Health Record	Actors: Study PI, Hospital leadership, IT Action: Build dashboard to capture *Code Sepsis* Activations in Electronic Health Record Action target: Usage of *Code Sepsis* Temporality: Preimplementation and Implementation phases Dose: Monthly meetings to develop the tools Implementation outcome affected: Service: efficiency, safety, clinical: Morbidity, mortality Justification: allow monitoring of *Code Sepsis* activation to track outcomes and provide feedback (see Audit & Provide Feedback below).
Audit & Provide Feedback (ERIC strategy)	Monthly review of SEP-1 outcomes at the hospital-wide Sepsis Steering Committee to provide feedback	Actors: Hospital-wide Sepsis Steering Committee Action: Review monthly SEP-1 outcomes from the HER dashboards Temporality: Implementation and Sustainment phases Dose: Monthly meetings Outcomes affected: Fidelity, Efficiency, safety, morbidity, mortality Justification: Tracking key metrics regularly will allow monitoring of compliance, feedback will lead to changes and progress toward outcomes

PI, principal investigator; RN, registered nurse; IT, Information Technology.

**Step 4: Select an Evaluation Framework to Measure Implementation Outcomes**

The final step in the design process is to select an *Evaluation Framework* to measure implementation outcomes. Evaluation frameworks provide resources to help examine the success of the implementation effort. An *Evaluation Framework* should be identified early in the project and will be used to guide outcome measurement when the implementation effort is underway. Implementation outcomes differ from efficacy/effectiveness outcomes in that they focus on the frequency and quality of intervention delivery rather than on clinical symptoms.^34^ For example, measuring implementation outcomes before and after the introduction of implementation strategies may focus on adherence to intervention components. Figure 1 shows the most common evaluation frameworks, which conceptualize specific implementation outcomes.

The 2 most used evaluation frameworks are RE-AIM^35^ and Proctor’s Implementation Outcomes Framework.^36^ Both have been cited over 2000 times and assist in conceptualizing and measuring implementation outcomes.^26^ Additionally, the CFIR Outcomes Addendum^37^ is a newer framework that organizes outcomes for use with the CFIR determinant framework.

The RE-AIM framework, which stands for *Reach, Effectiveness, Adoption, Implementation*, and *Maintenance*, is a public health framework that includes implementation and clinical outcomes. The RE-AIM website (reaim.org) thoroughly defines each variable and provides examples of applications and quantitative and qualitative measures for each outcome.

The 5 RE-AIM outcomes include:

• *Reach*: The proportion of individuals who are willing to receive the EBI, considering how many are eligible.
• *Effectiveness*: The impact of a program on individual outcomes.
• *Adoption* or first-time use by target recipients (e.g., staff, clinic): The absolute number, proportion, and representativeness of program deliverers who are willing to initiate a program.
• *Implementation*: Deliverer’s fidelity to the core components of a program, such as consistency of delivery as intended.
• *Maintenance*: The extent to which a program or policy becomes part of the organization’s routine practice and policies.

***Proctor Implementation Outcomes Framework***

The Proctor Implementation Outcomes Framework names 8 implementation outcomes used to evaluate implementation efforts:

• *Acceptability*: An individual’s (i.e., provider, patient) satisfaction with aspects of the program.
• *Adoption*: A provider or organization’s uptake or initial utilization of a program.
• *Appropriateness:* A provider, patient, or organization’s perceived fit of a program, such as its usefulness, compatibility, or practicability.
• *Feasibility:* A program’s compatibility and trialability with individual providers or an organization/setting.
• *Fidelity*: Whether the innovation is delivered as intended by an individual provider.
• *Implementation cost*: The provider’s or the organization’s cost and resources of implementation, including cost-effectiveness and marginal cost.
• *Penetration*: Similar to *Reach* in RE-AIM, measures the level of institutionalization and service access of a program.
• *Sustainability*: Similar to *Maintenance* in RE-AIM, measures an organization’s ability to continue a program as routine practice after some time.

***CFIR 2.0 Outcomes Addendum***

The CFIR 2.0 Outcomes Addendum proposes that certain implementation outcomes, such as *Feasibility*, *Acceptability*, and *Appropriateness*, should be considered implementation *antecedents* and measured earlier in the implementation process. In other words, if an intervention is not considered to be acceptable, appropriate, or feasible, it is unlikely to have strong *Adoption*, *Implementation*, or *Sustainment*, which are considered the true implementation outcomes.

**Case Example: Measuring Outcomes**

The *Code Sepsis* study used the Proctor Implementation Outcomes Framework^36^ as a guide for evaluating the effectiveness of the implementation strategies and providing insights for future improvements. Table 2 shows the Proctor outcomes and the *Code Sepsis*-specific definition for each outcome. The “Outcomes” column of the IRLM (Fig 2) defines the implementation, service, and clinical outcomes. Here, the service outcomes were efficiency (eg, time to receive treatment), safety (eg, fewer errors, overall compliance with the bundle components), and the clinical outcomes were reducing patient morbidity and mortality.

**Table 2:** *Code sepsis* implementation outcomes according to the Proctor Implementation Outcomes framework.

Proctor Implementation Outcome	Code Sepsis definition
Acceptability	The extent to which Code Sepsis is perceived as agreeable, palatable, or satisfactory.
Adoption	The intention, initial decision, or action to try or employ Code Sepsis
Appropriateness	The perceived fit, relevance, or compatibility of Code Sepsis in the ED.
Feasibility	The extent to which Code Sepsis can be successfully used or carried out within the ED
Fidelity	The degree to which Code Sepsis was implemented as intended by the developer
Penetration	The number of eligible patients receiving Code Sepsis divided by the total number of patients eligible for Code Sepsis
Sustainability	The extent to which Code Sepsis is maintained or institutionalized within the ED’s ongoing, stable operations.
Cost	The cost impact of Code Sepsis implementation effort, based on the costs of Code Sepsis, the implementation strategy used, and the location of service delivery

ED, emergency department.

***Code Sepsis* Progression**

After implementation, the *Code Sepsis* implementation team conducted an informal determinant assessment and learned that the initial education helped clinicians better appreciate the importance of early sepsis recognition and intervention. Although people generally liked the concept of *Code Sepsis*, the workflow did not always function as intended in practice. The biggest issue was that the expected resources, such as a second registered nurse or other team support, were not always reliably available. There were also gaps in staff knowledge due to a high turnover rate and rate of contract staffing. That mismatch between the intended protocol and real-world execution generated frustration and contributed to a drop-off in enthusiasm after the initial rollout. Because of that feedback, the team identified a set of implementation strategies better aligned to identified barriers. Specifically, new strategies include buy-in from nursing and physician leadership (*involve local opinion leaders*) and puts more emphasis on operational reliability (*develop and implement tools for quality monitoring*). One facilitator to the enhanced high-level buy-in is that sepsis bundle compliance has become a mandatory pay-for-performance measure on the CMS Hospital Value-Based Purchasing Program, which has led to increased institutional prioritization of improving performance on this metric. Another strategy (*conduct educational meetings*) focuses frontline education much more on the “why.” Rather than emphasizing bundle compliance, the “why” is that sepsis is a major cause of inhospital mortality; many patients who die with sepsis have it present on admission, and early recognition and timely antibiotics are the most actionable steps clinicians can take in the ED. This approach to determinant identification and selection of matching strategies is expected to have a positive impact on sepsis bundle compliance and reductions in patient mortality.

## Conclusion

The Implementation Science schematic diagram for EP leaders can serve as a guide to support the translation of innovations into practice, particularly in the fast-paced and highly burdened emergency medicine setting. Each step incorporates commonly used TMFs that also accommodate the emergency medicine setting. Future directions include using these steps to carry out implementation studies and projects and conducting systematic reviews to determine characteristics of helpful strategies in the emergency medicine setting.

## Author Contributions

Emily S. Fu: Conceptualization; Primary authorship of the paper; Review and editing of the paper. Linda E. Guzman: Conceptualization; Review and editing of the paper. Yosef Berlyand: Conceptualization; Review and editing of the paper. Stephanie Chow Garbern: Conceptualization; Review and editing of the paper. Debasree Banerjee: Conceptualization; Review and editing of the paper. Kristine Durkin: Conceptualization; Review and editing of the paper. Hannah E. Frank: Conceptualization; Primary authorship of the paper; Review and editing of the paper; Review supervision, Visualization.

## Funding and Support

By *JACEP Open* policy, all authors are required to disclose any and all commercial, financial, and other relationships in any way related to the subject of this article as per ICMJE conflict of interest guidelines (see www.icmje.org). ESF is supported by the University of Chicago Primary Care Investigators Training in Chronic Disease & Health Disparities (PITCH) Fellowship (Health Resources and Services Administration T32 HP42019). LEG is funded by 10.13039/100000050NHLBI (T32HL176428). The funding body did not have a role in the design of the study, the collection, analysis, and interpretation of the data, or writing the manuscript.

## Conflict of Interest

All authors have affirmed they have no conflicts of interest to declare.
