# Supplementary material for: Implementation Science for Emergency Physicians: A Schematic Diagram and Step-By-Step Guidance to Implement Evidence-Based Innovations
Source: J Am Coll Emerg Physicians Open. 2026 Aug 27;7(5):100501. doi: 10.1016/j.acepjo.2026.100501 (PMC13545714; doi:10.1016/j.acepjo.2026.100501)
Supplement: Supplementary file 1 [file mmc1.docx]

***Appendix 1***. Template of the Schematic Diagram
